# Prevalence and Economic Impact of Acute Respiratory Failure in the Prehospital Emergency Medical Service of the Madrid Community: Retrospective Cohort Study

**DOI:** 10.2196/66179

**Published:** 2025-01-16

**Authors:** Ana María Cintora-Sanz, Cristina Horrillo-García, Víctor Quesada-Cubo, Ana María Pérez-Alonso, Alicia Gutiérrez-Misis

**Affiliations:** 1 Servicio de Urgencias Médicas de Madrid (SUMMA112), Universidad Autónoma de Madrid, Madrid, Spain; 2 Servicio de Urgencias Médicas de Madrid (SUMMA112), Ventilation Commission, Calle Antracita S/N, Madrid, Spain, 34 913387555; 3 Technical Health Research, Madrid, Spain; 4 Servicio de Urgencias Médicas de Madrid (SUMMA112), Research Commission, Madrid, Spain; 5 Department of Medicine, Division of Family Medicine and Primary Care, Clinical Simulation Laboratory, Faculty of Medicine, Universidad Autónoma de Madrid, Madrid, Spain

**Keywords:** acute respiratory failure, COVID-19, chronic obstructive respiratory insufficiency, congestive heart failure, bronchospasm, emergency medical services costs, ambulances, SARS-CoV-2, coronavirus, respiratory, pulmonary, pandemic, economic impact, observational, Madrid, community, medical records, health records, medical advanced life support, ALS, acute pulmonary edema, chronic obstructive pulmonary disease, COPD, prevalence

## Abstract

**Background:**

Chronic obstructive pulmonary disease (COPD), congestive heart failure (CHF), and acute pulmonary edema (APE) are serious illnesses that often require acute care from prehospital emergency medical services (EMSs). These respiratory diseases that cause acute respiratory failure (ARF) are one of the main reasons for hospitalization and death, generating high health care costs. The prevalence of the main respiratory diseases treated in a prehospital environment in the prepandemic period and during the COVID-19 pandemic in Spain is unknown. The Madrid Community EMS is a public service that serves all types of populations and represents an epidemiological reference for supporting a population of 6.4 million inhabitants. The high volume of patients treated by Madrid’s medical advanced life supports (ALSs) allows us to analyze this little-studied problem.

**Objectives:**

Our goal was to lay the groundwork for comprehensive data collection and surveillance of respiratory failure, with an emphasis on the most prevalent diseases that cause it, an aspect that has been largely overlooked in previous initiatives. By achieving these objectives, we hope to inform efforts to address respiratory failure and establish a standardized methodology and framework that can facilitate expansion to a continuous community-wide registry in Madrid, driving advances in emergency care and care practices in these pathologies. The aim of this retrospective observational study was to determine the pathologies that have mainly caused respiratory failure in patients and required medicalized ALS and to evaluate the cost of care for these pathologies collected through this pilot registry.

**Methods:**

A multicenter descriptive study was carried out in the Madrid Community EMS. The anonymized medical records of patients treated with medical ALS, who received any of the following medical diagnoses, were extracted: ARF not related to chronic respiratory disease, ARF in chronic respiratory failure, exacerbations of COPD, APE, CHF, and bronchospasm (not from asthma or COPD). The prevalence of each pathology, its evolution from 2014 to 2020, and the economic impact of the Medical ALSs were calculated.

**Results:**

The study included 96,221 patients. The most common pathology was exacerbation of COPD, with a prevalence of 0.07% in 2014; it decreased to 0.03% in 2020. CHF followed at 0.06% in 2014 and 0.03% in 2020. APE had a prevalence of 0.01% in 2014, decreasing to 0.005% in 2020 with the pandemic. The greatest economic impact was on exacerbation of COPD in 2015, with an annual cost of €2,726,893 (which equals to US $2,864,628).

**Conclusions:**

COPD exacerbations had the higher prevalence in the Madrid region among the respiratory diseases studied. With the COVID-19 pandemic, the prevalence and costs of almost all these diseases decreased, except for ARF not related to chronic disease. The cost of these pathologies over 5 years was €58,791,031 (US $61,832,879).

## Introduction

Chronic obstructive pulmonary disease (COPD) is a public health epidemic. The current state of the art displays a high prevalence and high burden [1]. COPD is the fourth leading cause of death worldwide, accounting for 3.5 million deaths in 2021, approximately 5% of all global deaths [2]. Although systematic reviews on the prevalence of COPD at the hospital level have been published previously [3,4], the literature on current prevalence estimates of COPD exacerbations, treated in prehospital emergency medical services (EMSs), is sparse. In particular, there are few published data on differences in COPD prevalence between rural and urban areas and between countries [5-7]. In this context, relevant and timely information on COPD prevalence in this context is essential to inform, develop, and implement context-appropriate policies and programs for the prevention and optimal control of COPD exacerbations.

Congestive heart failure (CHF) is another highly prevalent disease with significant morbidity and mortality worldwide [8]. There are geographical variations in the epidemiology of CHF [9]. There is a substantial lack of data from the out-of-hospital setting as well, where CHF has different evolution depending on the acute treatment administered [10,11]. Acute pulmonary edema is associated with high mortality. It requires emergency management and usually admission to hospital [7,12,13]. Prospective studies have shown that early treatment applied by prehospital EMS improves short time outcomes and lower hospital readmissions, but the population susceptible to early treatment is unknown [14,15].

COPD, CHF, and acute pulmonary edema (APE) are serious illnesses that often require acute care from EMS [5,6,16]. Respiratory diseases that cause acute respiratory failure (ARF) are one of the main reasons for hospitalization [7,17], generating high health care costs [18,19]. These diseases include bronchospasm as well [20]. Studies on the prevalence and expenditures of prehospital EMSs are scarce [5,21-23]. Consequently, more precise epidemiological data are needed to address these issues and obtain a more accurate picture of the amount of care and the costs generated by these pathologies [24,25] in the prehospital EMS setting.

The main objective of the Acute Respiratory Failure Treated by the Prehospital Medical Emergency Service of Autonomous Community of Madrid (SUMMIRA) project is to determine the prevalence of the most frequent respiratory diseases assisted by the Autonomous Community of Madrid’s (CAM) advanced life supports (ALSs). The secondary objective is to measure the EMS economic cost of health care for patients. We present the following article in accordance with the STROBE (Strengthening the Reporting of Observational studies in Epidemiology) reporting checklist (Multimedia Appendix 1).

## Methods

### Study Design

The SUMMIRA study is an observational, quantitative, multicenter, and cross-sectional study of patients attended by the EMS of the CAM for 6 years.

### Sample Size

Between 2014 and 2020, 583,984 persons of the population of the Madrid Community were clinically treated by the CAM ALSs, after a triage in the 112 health emergency telephone number. Based on these medical examinations, the subjects were included in the study. A cohort of 22,085 subjects was identified as having decompensated COPD; 22,085 with a main diagnosis of CHF; 4676 with APE; 17,843 presented ARF not related to chronic respiratory failure (CRF); 6921 were diagnosticated with bronchospasm; and 17,851 were related with ARF in CRF. This study involved a total of 96,221 patients included from the electronic medical records (EMRs).

### Study Setting

The CAM has a population of 6.4 million inhabitants from 2014, both urban and rural areas. The CAM has 47 ALSs [26]. The ALSs are responsible for (1) emergency care, namely, patient care at home or on public roads in the Madrid Community and (2) interhospital transfers. The CAM is also in charge of the Helicopter Emergency Medical Service.

Each emergency care is provided by a health professional team formed by an emergency physician, an emergency nurse, and 1 or 2 emergency technicians; these teams assist at home and other places and take care of the patient in the mobile intensive critical unit (ALS).

The EMRs analyzed are those performed by the ALS physicians who attended the pathologies under study. These are the patients attended by the ALS of the Community of Madrid after the patients or their families called 112 (both in the city and in the outskirts).

The EMRs are located in a program named Integrated Management System of the Coordinating Center [27]. Integrated Management System of the Coordinating Center is a highly secure system of clinical data that has received urgent health care from the ALSs of the CAM.

### Ethical Considerations

The study was conducted in accordance with the Declaration of Helsinki. This study was reviewed and approved by the Madrid Regional Ethics Committee on Medication Research (dated: June 6, 2022/N° V8.2022). All data used in this study have been deidentified or anonymized to protect the privacy of participants. No information is available that would allow for the direct or indirect identification of individuals. The Madrid Regional Ethics Committee approval covers secondary analysis without additional consent.

Since the study involved the secondary analysis of previously collected data, the ethics committee determined that the project was exempt from further review under the category of anonymized, preexisting data analysis. The study complied fully with international ethical standards and the protection of participants’ rights.

All EMRs in this study have been anonymized to protect the privacy of participants. Rigorous measures were taken to ensure the confidentiality of the data and the integrity of the information collected complying, therefore, with the principles regarding data processing referred to in Article 5 of European Union Regulation 2016/679 of the European Parliament and Council of April 27, 2016 (General Data Protection Regulation).

### Sampling and Recruitment Procedure

#### Inclusion Criteria

Patients with EMRs were treated by the CAM ALSs who, according to usual clinical practice, had been diagnosed with ARF not related to CRF, exacerbation of chronic obstructive pulmonary disease (ECOPD), CHF, bronchospasm, or APE according to the codes of the *International Classification of Diseases, Ninth Revision* [9] between 2014 and 2020. Medical diagnostic criteria based on clinical and historical data were followed. The population residing in the CAM per year was taken as the reference population, according to the Spanish National Institute of Statistics [28].

The diagnoses of APE and ECOPD were defined as a “clinical picture” in the prehospital and hospital emergency manuals [29-31], with consensus documents [32,33] and systematic review such as by Bello et al [34] . The diagnostic criteria for COPD and APE are described in Multimedia Appendix 2 and Table 1. The other pathologies were diagnosed following the diagnostic criteria of the 12 Octubre Physician Manual [35] in accordance with another prehospital emergency manual [36] and the Servicio de Urgencias Medicas de Madrid (SUMMA112; Prehospital Emergency Medical Service of Autonomous Community of Madrid) Physician Clinical Guide for Acute Heart Insufficiency (Multimedia Appendix 3).

**Table 1.** Prevalence of congestive heart failure, exacerbation of chronic obstructive pulmonary disease (ECOPD), acute pulmonary edema, and bronchospasm acute respiratory insufficiency not related to chronic respiratory failure, of Madrid region population, based to the Madrid Community census [37], and the prehospital emergency medical service´s electronic medical record of Madrid from 2014 to 2020 (n=583,984).

Diseases^a^	2014	2015	2016	2017	2018	2019	2020
Congestive heart failure, n (%)	3593 (0.056)	3696 (0.057)	3651 (0.056)	3484 (0.054)	2895 (0.044)	2828 (0.042)	1938 (0.029)
ECOPD, n (%)	4415 (0.068)	4463 (0.069)	4112 (0.064)	4284 (0.066)	3935 (0.060)	3616 (0.054)	2020 (0.030)
Acute pulmonary edema, n (%)	819 (0.013)	800 (0.012)	725 (0.011)	711 (0.011)	652 (0.010)	616 (0.009)	353 (0.005)
Acute respiratory insufficiency in chronic respiratory failure, n (%)	2872 (0.044)	3034 (0.047)	2834 (0.044)	2797 (0.043)	2399 (0.036)	2362 (0.035)	1553 (0.023)
Bronchospasm, n (%)	1000 (0.015)	1104 (0.017)	1157 (0.018)	1157 (0.018)	1062 (0.016)	979 (0.015)	462 (0.007)
Acute respiratory insufficiency (not related to chronic respiratory failure), n (%)	2648 (0.041)	2736 (0.043)	2717 (0.042)	2573 (0.040)	2349 (0.036)	2289 (0.034)	2531 (0.038)

a Results from the retrospective observational research Acute Respiratory Failure Treated by the Prehospital Medical Emergency Service of Autonomous Community of Madrid (SUMMIRA).

#### Exclusion Criteria

**Figure 1.**
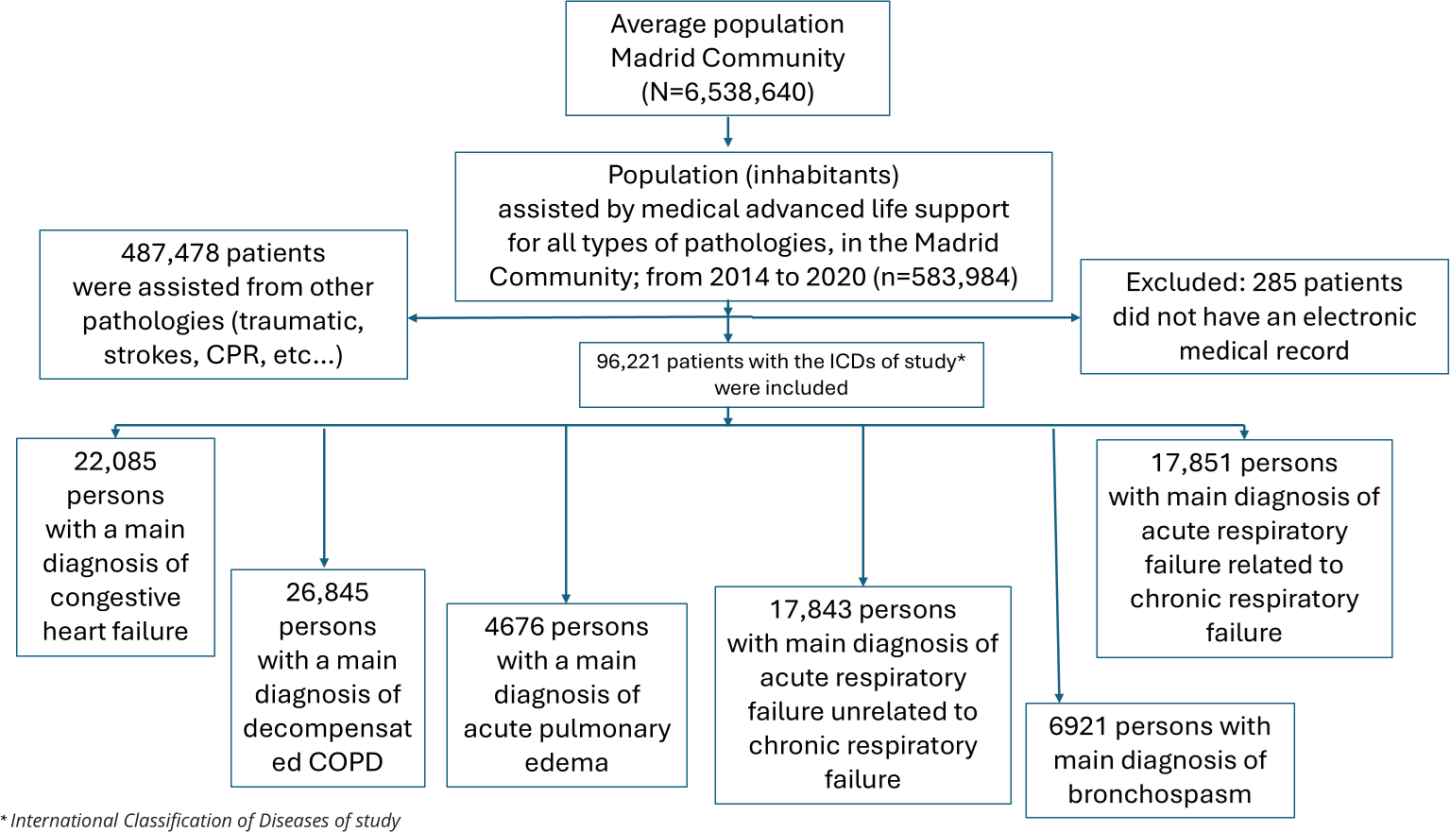
Cohort selection of patients assisted by the Madrid Community emergency medical service from January 1, 2014, to December 31, 2020. Acute Respiratory Failure Treated by the Prehospital Medical Emergency Service of Autonomous Community of Madrid (SUMMIRA) Study. COPD: chronic obstructive pulmonary disease.

Patients without a health identification number or without an adequate record in the basic minimum dataset were excluded. All patients diagnosed with asthma were excluded since they could be included among the patients with ARF related to chronic respiratory disease (Figure 1).

### Economic Analysis

The prevalence for each pathology was calculated by dividing the total number of individuals who presented the corresponding medical diagnosis in a given year by the population of Madrid at that time, according to the Spanish National Institute of Statistics.

The direct economic cost of CAM EMS care for the pathologies treated was calculated from the expenditure according to public references [38]. The health care cost data were extracted from the public prices for the provision of public health services and activities of the ALSs published in the Official Gazette of the CAM on August 21, 2017 (price list with an application date until December 2023). The reference cost for this study was the average cost of €611 (US $64,261), which is the arithmetic mean of mobilizing and assisting a patient by a terrestrial ALS unit in the public EMS of the Madrid Community [39]. The ALS provides advanced care, as it contains all the electromedical material and personnel that are required to form a mobile intensive care unit. The cost of ALS care (emergency medical services costs) includes physician care, emergency nursing assistance, and all required ventilatory support techniques, depending on the severity of the patient’s condition, with pharmacological treatment, oxygen therapy, invasive and noninvasive mechanical ventilation, and transfer to the hospital.

The direct economic impact was obtained by multiplying the cost of care for each pathology treated in the CAM’s ALSs by the number of times care was required. All persons within the CAM were able to receive care, as it is included in the universal emergency health care coverage of the Madrid Health Regional Government (public administration). The economic analysis was done in Microsoft Excel 365 and SPSS software (version 26; IBM Corp), with a copyright license from the Madrid Health Regional Government.

## Results

Table 1 shows a total of 96,221 patients who were included between 2014 and 2020. The most prevalent pathology among all those studied was COPD, with an initial prevalence of 0.068% (4415/6,454,440) in 2014, which decreased to 0.030% (2020/6,779,888) in 2020. CHF had a prevalence of 0.056% (3593/6,454,440) in 2014, decreasing to 0.029% (1938/6,779,888) in 2020. The prevalence of ARF in CRF was 0.044% (2872/ 6,454,440) in 2014 but decreased to 0.023% (1553/6,779,888) in 2020. The prevalence of bronchospasm (not from asthma or COPD) decreased from 0.015% (1000/6,454,440) in 2014 to 0.007% (462/6,779,888) in 2020. APE’s prevalence decreased from 0.013% (819/6,454,440) in 2014 to 0.005% (353/6,779,888) in 2020. The prevalence of ARF in patients not related to CRF decreased from 0.041% ( 2648/6,454,440) in 2014 to 0.034% (2289/6,663,394) in 2019 and increased to 0.038% (2531/6,779,888) in 2020, reversing this trend of ARF observed, especially in CHF and ECOPD.

The prevalence of APE shows a decreasing pattern from the onset. The other pathologies start with an upward trend, although in the second year, they reverse the trend and gradually decrease. The prevalence of bronchospasm maintains an upward trend until 2017 and from then on it starts to decrease progressively. In 2020, a sharper peak of decrease is assessed in all pathologies, except ARF not related to CRF, the latter being the only one to increase.

**Figure 2.**
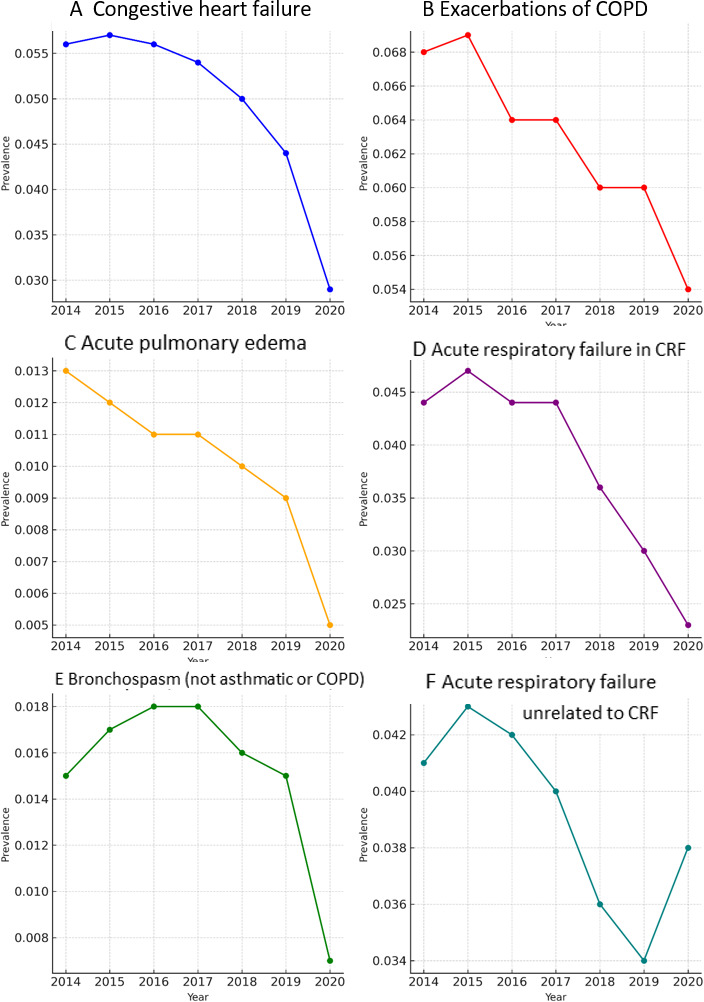
Changes in the prevalence of (A) congestive heart failure, (B) exacerbation of COPD, (C) acute pulmonary edema, (D) acute respiratory failure in chronic respiratory failure, (E) bronchospasm, and (F) acute respiratory insufficiency not related to chronic respiratory failure based on electronic medical record of the prehospital Madrid Community emergency medical service (2014-2020), assisted by prehospital emergency medical services. Results from the retrospective observational research Acute Respiratory Failure Treated by the Prehospital Medical Emergency Service of Autonomous Community of Madrid (SUMMIRA). COPD: chronic obstructive pulmonary disease; CRF: chronic respiratory failure.

A decrease in most respiratory diseases (Table 2) was observed in 2020, both for patients with ECOPD, APE, or bronchospasm (nonasthmatic, non-COPD) and for patients with CHF (Figures2A-F  3), except for ARF not associated with CRF, which corresponded in time and form with the COVID-19 pandemic period [40]. From 2017 on, a decrease in all types of ARF was observed, especially CHF and COPD.

Table 2 shows the economic cost of each pathology and its economic impact in each year. The greatest economic impact was observed in 2015 for ECOPD, with an annual cost of €2,726,893 (US $2,867,982). The estimated cost of all pathologies over 5 years was €58,791,031 (US $61,832,879).

**Table 2.** Direct economic impact of the congestive heart failure, exacerbation of chronic obstructive pulmonary disease (ECOPD), acute pulmonary edema (APE), bronchospasm, and acute respiratory failure not related to chronic respiratory failure of the Madrid region population; based on the prehospital Madrid emergency medical service’s electronic medical records^a^.

*ICD-CM*^b^ type	2014	2015	2016	2017	2018	2019	2020
Congestive heart failure							
Cases	3593	3696	3651	3484	2895	2828	1938
Cost (€)	2,195,323	2,258,256	2,230,761	2,128,724	1,768,845	1,727,908	1,184,118
ECOPD							
Cases	4415	4463	4112	4284	3935	3616	2020
Cost (€)	2,697,565	2,726,893	2,512,432	2,617,524	2,404,285	2,209,376	1,234,220
APE							
Cases	819	800	725	711	652	616	353
Cost (€)	500,409	488,800	442,975	434,421	398,372	376,376	215,683
Acute respiratory failure in chronic respiratory failure							
Cases	2872	3034	2834	2797	2399	2362	1553
Cost (€)	1,754,792	1,853,774	1,731,574	1,708,967	1,465,789	1,443,182	948,883
Bronchospasm							
Cases	1000	1104	1157	1157	1062	979	462
Cost (€)	611,000	674,544	706,927	706,927	648,882	598,169	282,282
Acute respiratory failure (not related to chronic failure)							
Cases	2648	2736	2717	2573	2349	2289	2531
Cost (€)	1,617,928	1,671,696	1,660,087	1,572,103	1,435,239	1,398,579	1,546,441
Total cost (€58,791,031) (€)	9,377,017	9,673,963	9,284,756	9,168,666	8,121,412	7,753,590	5,411,627
Total cost (US $61,832,879) (US $)	9,862,184	10,174,494	9,765,149	9,643,053	8,541,614	8,154,761	5,691,625

a Database from 2014 to 2020 included. Results from the retrospective observational research Acute Respiratory Failure Treated by the Prehospital Medical Emergency Service of Autonomous Community of Madrid (SUMMIRA).

b *ICD-CM*: *International Classification of Diseases, Clinical Modification*.

**Figure 3.**
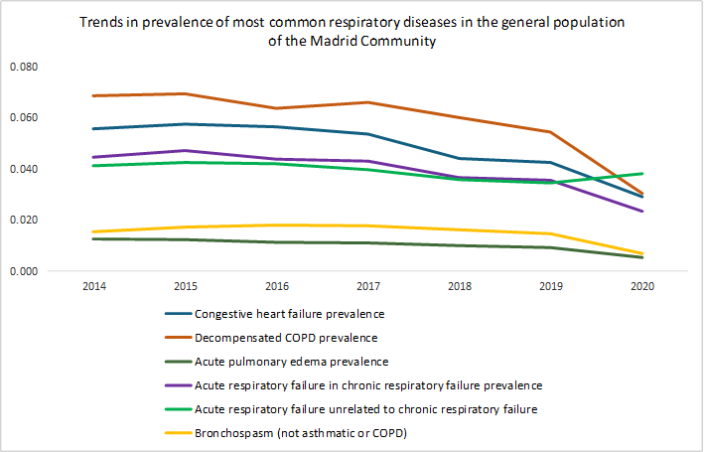
Evolution of congestive heart failure, exacerbation of chronic obstructive pulmonary disease, acute pulmonary edema, and acute respiratory failure in chronic respiratory failure prevalences in the Madrid region, based on the general population electronic medical records, assisted by prehospital emergency medical services, from 2014 to 2020. Results from the retrospective observational research Acute Respiratory Failure Treated by the Prehospital Medical Emergency Service of Autonomous Community of Madrid (SUMMIRA). COPD: chronic obstructive pulmonary disease.

## Discussion

### Principal Findings

Among the findings, we can mention that to our knowledge this is the only study in the prehospital EMS that has analyzed the prevalence of these pathologies in Spain and in Europe, so it can serve as a reference for future European studies on this topic. It was measured by the costs categorized by diagnosis. Finally, it is worth mentioning the large sample size, which provides greater reliability to the study. Few studies have quantified the direct costs incurred by ALSs in these pathologies [25,41-45]. A study carried out in Sweden and Belgium quantified a type of direct cost, such as transport for ECOPD, at 3% of the total direct costs [42]. In France, the cost of medical transport per patient was estimated to be €192‐273 (US $202-287) in 1996 [46]. In this study, the cost of air ambulance transportation, but not that of emergency medical care, was estimated.

In the ALS of the CAM, the demand by patients with dyspnea as the main diagnosis among patients with ECOPD was known, although there were no data to differentiate among ECOPD, bronchospasm, and other processes. Therefore, we needed to accurately calculate the prevalence of ARF in ALS to more precisely estimate the economic impact of these pathologies and their complete clinical picture, which includes emergent prehospital care [47].

The results of the SUMMIRA project have made it possible to estimate the global quantification for prehospital emergency care due to SARS-CoV-2 in the CAM, served by the ALSs of the CAM. The pathologies studied showed a continuously decreasing trend throughout the period analyzed, coinciding with the mortality data related to ECOPD published in the CAM [22]. The only different trend was the peak produced by ARF not associated with chronic failure, which, despite showing a decreasing trend from 2014 to 2019, increased the number of cases in 2020 at the same time as the COVID-19 pandemic in Spain [48].

These other declines previous to 2020 may be the result of many factors, such as regional success of tobacco control measures, improved information and prevention of allergens, better treatment of respiratory and cardiac comorbidities, and a reduction of universally high rates of underdiagnosis of chronic respiratory diseases [25]. The decreasing trend in the number of cases of most of the chronic respiratory diseases studied, such as COPD, CHF, and APE, before 2020, led us to estimate an improvement in disease control in chronic patients to date and a subsequent influence of the cases due to the COVID-19 pandemic [49].

The economic impact of chronic diseases on patients has not been quantified in the most recent COPD studies [7,21,50], as only primary care and hospital expenditure have been taken into account [22,45,51]. A descriptive North American study lasting 5 years with a large sample (166,908 patients) in which patients with respiratory distress transferred to the hospital was analyzed [6], but the population prevalences were not calculated.

Furthermore, there is a comparative dilemma between the different activities of the EMS’s ALSs in different countries. Some of them in Europe, such as the paramedical system, cannot discharge patients since transfer to hospital is part of the work protocol because some of them do not have doctors within health care teams who can discharge patients [52-54]. In this sense, the Spanish system of EMS [55] helps prevent care overload in hospitals since the physicians of ALSs allow patients to remain at home and can be treated there, improving adequately after receiving treatment and thus achieving benefits for both patients and the health system. Considering the number of cases of the diseases found, it would be advisable to evaluate the prevalence of these diseases in other similar European and national emergency health systems to have an updated global vision.

The set of decreasing trends in the prevalence of ECOPD is in line with the trend in industrialized countries [56]. Plausible causes include the greater control of adverse risk factors, as well as the increase in public health measures that protect against respiratory diseases (tobacco, asbestos, protective measures in occupational risks, etc) [18,25,57,58].

### Limitations

This work had a shortcoming in the population sample. This cohort did not include the care provided to the patients who go directly to the hospital emergency department; it is a prehospital cohort. The cohort would be bigger if it would include the patients of the Madrid Municipal Emergency Assistance and Rescue Service (SAMUR), though the number of patients treated by them is minimal because their assistance is limited to the public thoroughfare of Madrid capital, and by regular form this type of patient is treated at home and primary care that is covered by SUMMA112.

We have separated the average cost of the assistance provided by the advanced terrestrial life support services of the CAM to estimate the economic cost. Advanced air life support has also treated some patients, which is valued in Table 3.

**Table 3.** Case study of congestive heart failure, exacerbation of chronic obstructive pulmonary disease (ECOPD), acute pulmonary edema, bronchospasm, and acute respiratory insufficiency not related to chronic respiratory failure of the Madrid region population per year treated by Servicio de Urgencias Medicas de Madrid Helicopter Emergency Medical Service (SUMMA112 HEMS), from 2014 to 2020^a^.

Condition	2015	2016	2017	2018	2019	2020
ECOPD	1 ECOPD	1 ECOPD	2 ECOPD	2 ECOPD	3 ECOPD	1 ECOPD
Acute respiratory failure related to chronic respiratory failure	1 Acute respiratory failure in chronic respiratory failure	2 Acute respiratory failure in chronic respiratory failure	2 Acute respiratory failure related to chronic respiratory failure	1 Acute respiratory failure related to chronic respiratory failure	2 Acute respiratory failure related to chronic respiratory failure	1 Acute respiratory failure related to chronic respiratory failure
Acute pulmonary edema	1 Acute pulmonary edema	1 Acute pulmonary edema	2 Acute pulmonary edema	1 Acute pulmonary edema	3 Acute pulmonary edema	N/A^b^
Congestive heart failure	1 Congestive heart failure	4 Congestive heart failure	2 Congestive heart failure	N/A	N/A	3 Congestive heart failure
Bronchospasm	N/A	1 Bronchospasm	N/A	1 Bronchospasm	2 Bronchospasm	N/A
Acute respiratory failure not related to chronic respiratory failure	N/A	2 Acute respiratory failure not related.to chronic respiratory failure	2 Acute respiratory failure not related to chronic respiratory failure	1 Acute respiratory failure not related to chronic respiratory failure	3 Acute respiratory failure not related to chronic respiratory failure	N/A

a As can be valued in the Official Gazette of the Madrid Council [38], the cost per medical helicopter intervention in this period time is €5746 (US $6043).

b N/A: not applicable.

Our study shows a reduction in the costs of CHF over the years in EMS. The decrease may be explained by factors such as reduced severity and better treatment of acute coronary syndrome [21]. We have not found any similar bibliography in the prehospital setting. The 2019 Heart Failure Association Atlas reported a heart failure prevalence ranging from ≤12 in Spain [51]. At the European level, there is considerable heterogeneity of data between European countries, ranging from an overall prevalence of 14 per 1000 in the Netherlands [24] to 39 in Slovenia, Lithuania, and Germany but with a common point: EMS use in heart failure is an independent predictor of 30-day mortality [50]. In exacerbations of COPD, the patients have a significant comorbidity burden and experience high rates of hospitalization and mortality [59]. It can lead to several conclusions in the area of public health: (1) the measures taken by primary and specialized care have been effective in control of exacerbations of this type of pathology, and furthermore (2) despite reducing costs, in seasonal outbreaks a greater investment [51] of costs would be needed to reinforce the resources that deal with the ECOPD in the prehospital emergency services.

This study opens the possibility of including these values in the database of the economic impact of chronic respiratory diseases such as COPD, CHF, and APE, which are currently not considered [6-8]. In addition to the registration of their prevalence in the different months of the year, an analysis of the needs required in public health can be made to make investment plans in the times of the year when there is a higher prevalence. Moreover, it could be taken into account when assessing the costs associated with SARS-CoV-2–related bronchospasm and ARF in prehospital critical care for future respiratory transmission pandemics.

### Conclusions

The results found in this study are in line with the general hospitalization downward trend in high-income countries of a decrease in the number of cases from 2014 to 2020 for the most common respiratory diseases treated in hospital emergencies [21,51,56]. The prevalence of ECOPD in the Madrid EMS was higher than the other respiratory diseases studied. In 2020, with the COVID-19 pandemic, a decrease in the prevalence and costs of the pathologies studied was observed, except for the prevalence of ARFs not related to chronic failure. The greatest economic impact was for ECOPD in 2015, with an annual cost of €2,726,893 (US $2,867,982). The estimated cost of these pathologies in 5 years was €58,791,031 (US $61,832,879).

## Supplementary material

10.2196/66179 Multimedia Appendix 1 STROBE (Strengthening the Reporting of Observational studies in Epidemiology) reporting checklist.

10.2196/66179 Multimedia Appendix 2 Exacerbation of chronic obstructive pulmonary disease and acute pulmonary edema diagnosis.

10.2196/66179 Multimedia Appendix 3 Congestive heart failure diagnosis.

## Abbreviations

ALS: advanced life support
APE: acute pulmonary edema
ARF: acute respiratory failure
CAM: Autonomous Community of Madrid
CHF: congestive heart failure
COPD: chronic obstructive pulmonary disease
CRF: chronic respiratory failure
ECOPD: exacerbation of chronic obstructive pulmonary disease
EMR: electronic medical record
EMS: emergency medical service
STROBE: Strengthening the Reporting of Observational studies in Epidemiology
SUMMA112: Servicio de Urgencias Medicas de Madrid (Prehospital Emergency Medical Service of Autonomous Community of Madrid)
SUMMIRA: Acute Respiratory Failure Treated by the Prehospital Medical Emergency Service of Autonomous Community of Madrid
