# Supplementary material for: Prevalence and Economic Impact of Acute Respiratory Failure in the Prehospital Emergency Medical Service of the Madrid Community: Retrospective Cohort Study
Source: JMIR Public Health Surveill. 2025 Jan 16;11:e66179. doi: 10.2196/66179 (PMC11756833; doi:10.2196/66179)
Supplement: Multimedia Appendix 1 [file publichealth-v11-e66179-s001.docx]

STROBE Statement—checklist of items that should be included in reports of observational studies

| **Section/item** | **Item No** | **Recommendation** | **Reported on Page Number/Line Number** | **Reported on Section/Paragraph** |
| --- | --- | --- | --- | --- |
| **Title and abstract** | 1 | (a) Indicate the study’s design with a commonly used term in the title or the abstract | pg2, line38 | Abstract, paragraph 2 |
|  |  | (b) Provide in the abstract an informative and balanced summary of what was done and what was found | Pg3, line1 44-55 | Methods, paragraph 1  Results, paragraph 2 |
| **Introduction** | | | | |
| Background/ rationale | 2 | Explain the scientific background and rationale for the investigation being reported | pg5, line 81-89 pg5, line137-151 | Introduction, Paragraph 1  Methods, Paragraph 1 |
| Objectives | 3 | State specific objectives, including any prespecified hypotheses | pg 5, line 99-101 | Introduction, Paragraph 4 |
| **Methods** | | | | |
| Study design | 4 | Present key elements of study design early in the paper | pg5, line106-117 | Methods, Paragraph 2-3 |
| Setting | 5 | Describe the setting, locations, and relevant dates, including periods of recruitment, exposure, follow-up, and data collection | p5, line 90-98, 119-124 | Introduction paragraph 2 Methods, Paragraph 2-3 |
| Participants | 6 | (a) ***Cohort study***—Give the eligibility criteria, and the sources and methods of selection of participants. Describe methods of follow-up  ***Case-control study***—Give the eligibility criteria, and the sources and methods of case ascertainment and control selection. Give the rationale for the choice of cases and controls  ***Cross-sectional study***—Give the eligibility criteria, and the sources and methods of selection of participants | Pg 6, line142-147 | Methods, Paragraph 7 |
|  |  | (b) ***Cohort study***—For matched studies, give matching criteria and number of exposed and unexposed  ***Case-control study***—For matched studies, give matching criteria and the number of controls per case | Not a matched study | Not a matched study |
| Variables | 7 | Clearly define all outcomes, exposures, predictors, potential confounders, and effect modifiers. Give diagnostic criteria, if applicable | Pg 6, line 142-154 | Methods, Paragraph 8 |
| Data sources/ measurement | 8* | For each variable of interest, give sources of data and details of methods of assessment (measurement). Describe comparability of assessment methods if there is more than one group | Pg 20, line 315-337 | Annex 1-2 |
| Bias | 9 | Describe any efforts to address potential sources of bias | Pg18, line 155-157 | Methods Paragraph 9 |
| Study size | 10 | Explain how the study size was arrived at | Pg8, line153, figure 1 | Methods, Figure 1 |
| Quantitative variables | 11 | Explain how quantitative variables were handled in the analyses. If applicable, describe which groupings were chosen and why | Pge 5, line 106-115 | Methods Paragraph 1-2 |

| Statistical methods | 12 | (a) Describe all statistical methods, including those used to control for confounding | Pg5, line162-184 | Methods, Last Paragraph |
| --- | --- | --- | --- | --- |
|  |  | (b) Describe any methods used to examine subgroups and interactions | Pg5, line156-157 | Methods, Paragraph 9 |
|  |  | (c) Explain how missing data were addressed | pg8 line154-159 | Methods, Paragraph 2 |
|  |  | (d) ***Cohort study***—If applicable, explain how loss to follow-up was addressed  ***Case-control study***—If applicable, explain how matching of cases and controls was addressed  ***Cross-sectional study***—If applicable, describe analytical methods taking account of sampling strategy | pg8, line154-159 | Methods, Paragraph 2 |
|  |  | (e) Describe any sensitivity analyses | pg 8, line 161-171 | Methods, Paragraph 3 |
| **Results** | | | | |
| Participants | 13* | (a) Report numbers of individuals at each stage of study—eg numbers potentially eligible, examined for eligibility, confirmed eligible, included in the study, completing follow-up, and analysed | Figure 1, pg7, line153 | Methods, Figure 1 Paragrapgh 2 |
|  |  | (b) Give reasons for non-participation at each stage | Pg 5, line156 | Methods, Figure 1 |
|  |  | (c) Consider use of a flow diagram | Figure 1, pg7, line153 | Methods, Figure 1 |
| Descriptive data | 14* | (a) Give characteristics of study participants (eg demographic, clinical, social) and information on exposures and potential confounders | pg9-10, line186-199 | Results, Paragraph 1 |
|  |  | (b) Indicate number of participants with missing data for each variable of interest | Figure 1, pg7 | Methods, Figure 1 |
|  |  | (c) ***Cohort study***—Summarise follow-up time (eg, average and total amount) | not applicable | not applicable |
| Outcome data | 15* | ***Cohort study***—Report numbers of outcome events or summary measures over time | not applicable | not applicable |
|  |  | ***Case-control study***—Report numbers in each exposure category, or summary measures of exposure | not applicable | not applicable |
|  |  | ***Cross-sectional study***—Report numbers of outcome events or summary measures | pg10, line201-254 |  |
| Main results | 16 | (a) Give unadjusted estimates and, if applicable, confounder-adjusted estimates and their precision (eg, 95% confidence interval). Make clear which confounders were adjusted for and why they were included | not applicable | not applicable |
|  |  | (b) Report category boundaries when continuous variables were categorized | not applicable | not applicable |
|  |  | (c) If relevant, consider translating estimates of relative risk into absolute risk for a meaningful time period | not relevant | not relevant |
| Other analyses | 17 | Report other analyses done—eg analyses of subgroups and interactions, and sensitivity analyses | pg10, line201-254, table 2 | Economic analysis |
| **Discussion** | | | | |
| Key results | 18 | Summarise key results with reference to study objectives | Pg 9, line 258-272 | Discussion, Paragraph 6,7 |
| Limitations | 19 | Discuss limitations of the study, taking into account sources of potential bias or imprecision. Discuss both direction and magnitude of any potential bias | Pg10, line 276-283 | Limitations, paragraph 1,2 |

| Interpretation | 20 | Give a cautious overall interpretation of results considering objectives, limitations, multiplicity of analyses, results from similar studies, and other relevant evidence | Pg10, line 284-509 | Discussion, Paragraph 1 |
| --- | --- | --- | --- | --- |
| Generalisability | 21 | Discuss the generalisability (external validity) of the study results | pg23, line 298-302 | Limitations, last paragraph, Conclusions, paragraph 1 |
| **Other information** | | | | |
| Funding | 22 | Give the source of funding and the role of the funders for the present study and, if applicable, for the original study on which the present article is based | Pg 20, line 365/367  Pg 24, line 529/530 | Conflicts of Interest  Statements and Declarations |

*Give information separately for cases and controls in case-control studies and, if applicable, for exposed and unexposed groups in cohort and cross-sectional studies.

**Note:** An Explanation and Elaboration article discusses each checklist item and gives methodological background and published examples of transparent reporting. The STROBE checklist is best used in conjunction with this article (freely available on the Web sites of PLoS Medicine at [http://www.plosmedicine.org/,](http://www.plosmedicine.org/) Annals of Internal Medicine at [http://www.](http://www/) annals.org/, and Epidemiology at [http://www.epidem.com/).](http://www.epidem.com/)) Information on the STROBE Initiative is available at [www.strobe-statement.org.](http://www.strobe-statement.org/)

Article information

*As the checklist was provided upon initial submission, the page number/line number reported may be changed due to copyediting and may not be referable in the published version. In this case, the section/paragraph may be used as an alternative reference.
