# Supplementary material for: Prevalence and Economic Impact of Acute Respiratory Failure in the Prehospital Emergency Medical Service of the Madrid Community: Retrospective Cohort Study
Source: JMIR Public Health Surveill. 2025 Jan 16;11:e66179. doi: 10.2196/66179 (PMC11756833; doi:10.2196/66179)
Supplement: Multimedia Appendix 2 [file publichealth-v11-e66179-s002.docx]

**Appendix 2:** ECOPD & APE Diagnosis

**Table 1**: Clinical Diagnoses of Exacerbation of Chronic Obstructive Pulmonary Disease and Acute Pulmonary Edema. Source: Ventilation Manual in Prehospital EMS SUMMA112, 2022. [29]

| **Aspect** | **Acute Exacerbation of COPD** | **Acute Pulmonary Edema (APE)** |
| --- | --- | --- |
| **Definition** | Worsening of symptoms in a previously diagnosed COPD patient, characterized by acute or subacute respiratory deterioration. | Rapid onset of fluid accumulation in the lungs, typically due to cardiac issues. |
| **Main Cause** | Acute exacerbation of respiratory symptoms in a COPD patient, potentially triggered by infection or environmental factors. | Heart failure, leading to increased pressure in the pulmonary circulation and fluid leakage into the lungs. |
| **Main Symptoms** | Increased dyspnea (shortness of breath), worsening cough with increased sputum production (sometimes purulent), fever, tachypnea, cyanosis. | Sudden onset of dyspnea, orthopnea, paroxysmal nocturnal dyspnea, bilateral pulmonary crackles, and wheezing (cardiac asthma). |
| **Additional Signs** | Flapping tremor, hypoxemia, tachypnea, wheezing, rhonchi, use of accessory respiratory muscles, hypercapnia, possible hypoperfusion, cyanosis, and signs of right heart failure (in severe cases). | Heart rate >120 bpm, respiratory rate >25 rpm, retractions, oxygen saturation (SpO₂) <90%, bilateral crackles on lung auscultation. |
| **Diagnosis** | Clinical, based on a change in symptoms (semiology), especially respiratory signs in a known COPD patient. In non-diagnosed patients, it is considered as a possible COPD exacerbation. | Clinical, based on acute respiratory symptoms, auscultation findings, and SpO₂ measurements below 90%. |
| **Prognosis** | Depends on severity and timely treatment, with the risk of repeated exacerbations leading to further lung damage. | Serious and potentially life-threatening if not treated promptly. Dependent on managing underlying cardiac causes. |

**Extracted from**,

<Manual de Ventilación Mecánica del SUMMA112 https://www.comunidad.madrid/publicacion/ref/50596’Comunidad de Madrid. Accessed: June. 16, 2024. [Online]. Available: https://www.comunidad.madrid/publicacion/ref/50596>
