# Supplementary material for: Prevalence and Economic Impact of Acute Respiratory Failure in the Prehospital Emergency Medical Service of the Madrid Community: Retrospective Cohort Study
Source: JMIR Public Health Surveill. 2025 Jan 16;11:e66179. doi: 10.2196/66179 (PMC11756833; doi:10.2196/66179)
Supplement: Multimedia Appendix 3 [file publichealth-v11-e66179-s003.docx]

**Appendix 3: Congestive Heart Failure Diagnosis**

It is based on the Physician Clinical Guide of management and treatment of Acute Heart Failure in the Prehospital EMS SUMMA112

It is primarily clinical; it is established early in the pre-hospital setting to initiate appropriate physician treatment.

1• Symptoms. 2• Previous cardiovascular history. 3• Potential triggers, cardiac and non-cardiac.

1. Assessment of signs/symptoms of congestion or hypoperfusion. Symptoms and signs of acute heart failure reflecting fluid overload (pulmonary congestion or peripheral edema) and decreased cardiac output with peripheral hypoperfusion.

| - Acute left heart failure: |
| --- |
| - - (retrograde repercussions): Symptoms/signs of pulmonary congestion: −Exertional dyspnea − Orthopnea − Paroxysmal nocturnal dyspnea −Pulmonary crackles (bilateral) |
| - - (anterograde repercussion): Symptoms/signs of hypoperfusion − Cold, sweaty extremities − Oliguria – Mental confusion − Dizziness − Tight pulse pressure |
| - Acute right heart failure: - Symptoms/signs of congestion: − Jugular venous engorgement −Peripheral edema (bilateral) − Congestive hepatomegaly −Hepatojugular reflux − Ascites. |
